# Comparisons of stakeholders' influences, inter-relationships, and obstacles for circular economy implementation on existing building sectors

**DOI:** 10.1038/s41598-024-61863-0

**Published:** 2024-05-14

**Authors:** Sakdirat Kaewunruen, Patrick Teuffel, Ayfer Donmez Cavdar, Otso Valta, Tatjana Tambovceva, Diana Bajare

**Affiliations:** 1 Department of Civil Engineering, University of Birmingham, Birmingham, B15 2TT UK; 2 SRH Berlin School of Technology, Ernst-Reuter-Platz 10, 10587 Berlin, Germany; 3 Department of Forest Industry Engineering Faculty of Forestry, Karadeniz Technical University, 61080 Trabzon, Turkey; 4 Avoin Association, Helsinki, Finland; 5 BALTECH Study Centre, Faculty of Engineering Economics and Management, Riga Technical University, Kipsalas Street 6A, Riga, 1048 Latvia; 6 Institute of Materials and Structures, Faculty of Civil Engineering, Riga Technical University, Kipsalas Street 6A, Riga, 1048 Latvia

**Keywords:** Circular economy, Stakeholder engagement, Stakeholder influence, Interrelationship, Obstacles, Barriers, Circular buildings, Circular materials, Sustainable assessment, Business models, Life cycle perspective, Civil engineering, Sustainability

## Abstract

Buildings are energy- and resource-hungry: their construction and use account for around 39% of global carbon dioxide emissions; they consume around 40% of all the energy produced; they are responsible for over 35% of the EU's total waste generation; and account for about 50% of all extracted (fossil) materials. Therefore, they present a significant challenge to meeting national and international Net Zero targets of reducing greenhouse emissions and fossil resource use. The CircularB Project, is at the heart of this issue, which will underpin synergies of multi-scale circular perspectives (from materials, to components, to assets and built environments), digital transformation solutions, data-driven and complexity science, stakeholder behavioral science, and interdisciplinary capabilities towards achievable, affordable and marketable circular solutions for both new and existing buildings, for sustainable urban design, and for circular built environments across Europe. This paper contributes to the project by deriving new insights into the stakeholders’ influences, inter-relationships, and obstacles in the implementation of circular economy concepts on existing building stocks in Europe, which represent over 90% of whole building assets. In order to identify and derive the insights, our study is rigorously based on (i) a robust critical literature review of key documentations such as articles, standards, policy reports, strategic roadmaps and white papers; and (ii) interviews with relevant stakeholders and decision makers. Uniquely, our work spans across all scales of CE implementation from materials, to products and components, to existing building stocks, and to living built environments. The findings point out the current challenges and obstacles required to be tackled. Inadequacies of financial incentives and governmental enforcement (via policy, legislation, or directive) are commonly found to be the most critical obstacles found throughout Europe. Circular economy is the global challenge and not just a single country can resolve the climate issue without the cooperation of other countries. The insights thus highlight the essential need for harmonized actions and tactical/pragmatic policies promoted and regulated by the European Commission, national and local governments who can dominate the influence, promote inter-relationship, and overcome the barriers towards circular economy much more effectively.

## Introduction

The European Commission has recently reported that, by 2050, the world will exploit tripple of today’s resource demand. In the next 40 years, the world consumption of key materials such as biomass, fossil fuels, metals and minerals is expected to double, while waste generation is estimated to increase by 70%^1^. The resource demand exoponentially induces the economic activities, products and infrastructures, resulting in emormous greenhouse gas emission that is the root cause of climate change. The European Green Deal has thus launched a concerted strategy for a climate-neutral, resource-efficient and competitive economy^1^. In order to achieve climate neutrality by 2050, we need to scale up circular economy (CE) guidelines to the mainstream industry practices and economic instruments. This action will decouple economic growth from raw resource consumption, while ensure the long-term competitiveness of the EU and leaving no one behind. To fulfill this ambition, the EU develops the EU Green Deal to accelerate the transition towards a regenerative growth model that strives to reduce its consumption footprint and double its circular material use rate in the coming decade.

The applications of CE principles in real estate and building sectors are mostly restricted to new building stocks. This is because the circularity can be embedded and facilitated at the early design stage aiming for adaptability, modularity, durability, waste reduction and high-quality management. The early design of new building stocks can improve the whole life cycle of a building and its components by prolonging service lives, improving maintainability, increasing the ability to reuse, repurpose, and recycle, and minimizing energy consumption and wastes of materials, components and building assets at different stages of life cycle. In contrast, circular economy practices within existing buildings are not well established nor adequately implemented. The definitions of circular economy, and more specifically circularity in the built environment, are currently diverse, incoherent and unsystematic. This is because the purposes and goals to redevelop, revamp or renovate existing buildings at different ages (or service lives) can be highly varied. CE needs to be considered as a business strategy, and should not be viewed merely a waste management or a design strategy. Improving existing buildings' services and added values (e.g. nearly zero energy consumption, prolonged building components, removal of toxicity and pollution, ability and potential to reuse and even upcycling, etc.) should be rather be emphasised instead of only viewing those as potential material banks for downcycling. In fact, recovered materials and components from existing buildings face a critical barrier. Their direct reuse is skeptical due to various uncertainties in their technical compatibility, valorisation potential, and quality appraisal. This causes further downcycling processes and exacerbating extra resources and energy losses.

A key goal that generates most actions and activities within existing building stocks is to minimize energy consumption within the existing buildings (i.e. towards zero energy consumption). It has also been reported that 40% of total energy consumption came from buildings, which resonates with the US buildings accounting for 41% of their energy consumption and buildings all together accounted for 1/3 of the world’s energy use^1–3^. The need to combat the extensive energy use in buildings has been paramount and the EU imposed Energy Performance of Buildings Directive targets: all new *public* buildings had to be nearly zero-energy by 2018 and all new buildings had to be nearly zero-energy by the end of 2020. In October 2020, the European Commission (EC) published its new Renovation Wave Strategy to further improve the energy performance of *existing* buildings. It aims to double renovation rates in the next 10 years while ensuring that renovations lead to higher energy, circular economy, and resource efficiency^1–3^. Although existing building blocks are the majority of whole building stocks, the rate of renovation of existing buildings in Europe is currently between 1.2 and 1.4% per year and therefore the largest part of the European building stock continues to rely on a large extent on fossil fuels for its energy needs. For example, the share of the annual building stock that undergoes a major renovation can be: (i) below 1% in Spain, Poland, Italy or Sweden; (ii) around 1% in the Netherlands or Lithuania; (iii) above 1.5% in other countries like Germany, France or Austria^4–7^. This implies that the transitions to net zero are at risk due to the fact that challenges and barriers to implement circular built environments exist.

According to the European Commission, 35 million existing buildings could be renovated and up to 160,000 additional green jobs created in the construction sector by 2030. Such energy-efficiency renovations are deemed crucial for making Europe climate-neutral by 2050 according to Ref.^8^ and can accelerate the transition towards a regenerative growth model in accordance with the EU Circular Economy Action Plan. Embracing higher energy reduction innovations (through further circular design, retrofit and renovation strategies) is thus desperately needed to further address net zero energy, building lifecycle and climate change adaptation issues, and has the associated potential to timely benefit business and competitiveness for Europe in the digital era. To date, European and Member State sustainable targets have been pushed back due to the coronavirus pandemic and lack of variety of building energy reduction and affordable harvesting techniques suitable for particular stages of building lifecycle in European markets^9–12^. Reducing energy consumption is a top priority under the Energy Performance of Buildings Directive (EPBD) and the 2020 and 2050 objectives on energy efficiency. While the proposal places energy as one target, it is observed how European directives are the driver of designs and urban planning that neglects the climate change adaptation, digital transformation, and lifecycle design approaches^13–15^. Therefore, any new strategies to implement circular economy to existing building stocks will timely progress the transaction from the lack of any tailored design and optimal renovation methods to a new suite of adaptive and diverse circular re-design, retrofit, and renovation methods, which enrich co-values, regenerative circularity, lifecycle benefits, digital transformation, and relationships with the natural environment.

A range of global grand challenges have been identified at the core of the emerging global trends to 2050, including urbanization, population growth, inter/intra-national social disparities, demographic change, climate change, and ethics. The evidence of climate change, and its effects on legislative requirements, and market demands, has moved the circularity and energy independence agenda to an important and core position to act immediately in Europe^16–18^. The priority towards circularity, zero energy buildings, and beyond has been further amplified by low carbon targets which are high in the policy agenda after the ratification of the Paris Agreement. In contrast, a vast number of uncertainties, technology readiness, quality assurance, circularity and lifecycle issues have not been taken into account for in the past building performance and cost optimisation models, and these present a real market barrier and technology gap to be bridged. Within this broader context, a new EU Cost Action CircularB (https://circularb.eu/) has a key role to play since it has been described as a game changer, which can introduce major economic and social impacts e.g. potentials to reuse and recycle, energy consumption reduction, cash savings, fuel efficiency, novel cost-effective and resilient renovation technologies, new energy harvesting apparatus, and new circular business capabilities. While quantifying these positive impacts, optimal benefits have been estimated between 100 and 170 kWm/m^2^ annually per building over its life cycle (by the European Commission’s Building Directive)^19–21^.

Although every professional involved with the built environment sector attempts to embrace sustainability, circular economy and energy efficiency as the primary driver of their design, net zero has not been achieved at the European level^20,21,43^. This is evident by the annual deep energy renovation rate of merely 0.2% on average in the EU^20^. Efforts can be further encumbered by energy and environmental targets legislated under building design codes, which in their negotiation between ambitions and market readiness often default to the latter. Both EU Regulation and Voluntary Certification Systems focus on energy efficiency of the new buildings. However, the rate of new construction is only around 1% per year in Europe and therefore the highest potential for circular economy implementation and energy efficiency improvement lies in the existing building stocks (through deep renovation, retrofit, and repurpose). Social and economic growth, security and sustainability in Europe are therefore at risk of being compromised since existing building stocks have not been been sufficiently equipped with highly-efficent nearly zero energy building (NZEB) technologies. With increasing public demands for energy exposed to meta-operational uncertainties (under various extreme climate conditions), the building sector could fail to meet net zero target. In particular, the growing dependency of Europe on energy imports and expected high energy cost raise significant concerns on energy efficiency and the necessity for novel technologies to save and self-harvest energy within the built environment. Therefore, various actions for existing building stocks are commonly incentivised by the purpose towards energy efficiency. With this in mind, the implementation of circular economy practices in existing built environments can be further embeded to embrace on the detailed circular design upgrade for new, and retrofit and renovation of existing buildings and interconnected urban infrastructures for future cities, which are currently aging and inadequate. The existing building stocks will then reduce thier reliance on a large extent of energy and heat supply by fossil fuels, while having better capacity to enable regenerative growth through reusing, repurposing, recycling strategies. This study will thus review lessons learnt from the emerging sustainability and energy standards that are based on systems thinking approach and socio-technical systems. State-of-the-art reviews will therefore inform a new set of parameters and indicators that describe the higher levels of performance needed in both new and existing built environments of the future, and in so doing provide guidance, examples and an active and engaged community of experts and practitioners to deliver circular re-design, retrofit and renovation models and technology assemblies^22^.

**Figure 1:**
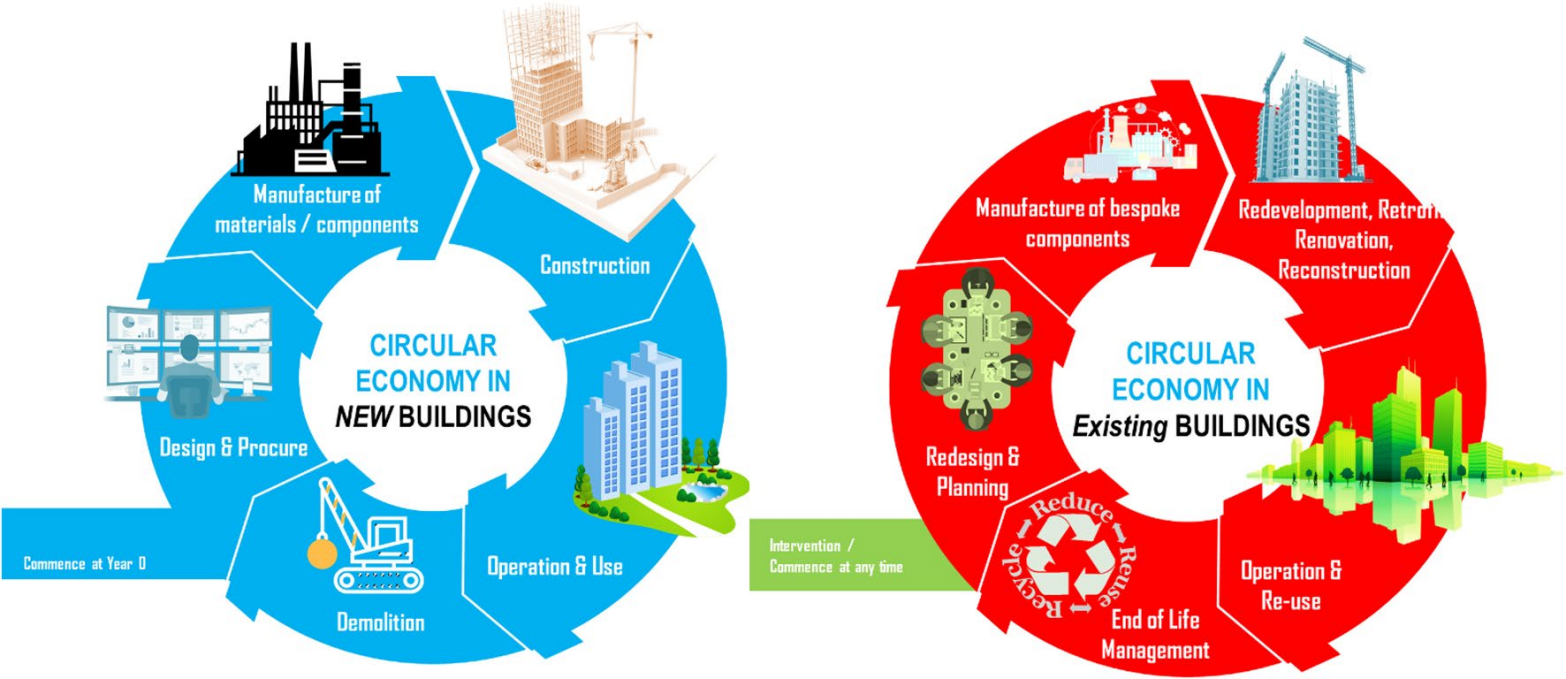
Comparison of lifecycle and circular economy implementation between new and existing building stocks.

The complexity of circular economy implementation for existing building stocks is much more pronounced in comparison to that for new buildings. This challenge is due to the fact that interventions into existing building stocks can occur at any stage of service lives (e.g. after 10 years; 20 years; or 50 years of usage), making it very difficult to attain an attractive value-based business case for circular intervention practices^23–27^. These aspects have raised not only the complexity but also the uncertainties in decision making and effective technical solutions that could seamlessly enable the transition to net zero. Figure 1 illustrates the difference between circular economy implementation to new and existing building stocks. It is clear that, when dealing with existing or aging building stocks, complex and refined scope of circular economy implementation is very evidential. The decision making mechanisms and influences among stakeholders become more delicate and personalized. This has raised a new challenge in developing pragmatic policies to promote and incentivize the adoption of circular economy perspective towards net zero.

This study aims to determine new insights into the stakeholders’ influences, inter-relationships, and obstacles in the implementation of circular economy concepts targeted at existing building stocks in Europe. The existing building stocks represent over 90% of whole building assets combined. On this ground, the insights in this study will be very critical and instrumental to the CE implementation and transition to net zero. In this study, the research methodologies are rigorously based on (i) a robust critical literature review of key documentations such as articles, standards, policy reports, strategic roadmaps and white papers; and (ii) interviews with relevant stakeholders and decision makers. The information in the first part will help to identify suitable questions for different stakeholders (to be interviewed in the later part). It is very clear from previous studies^4–7^ that existing building stocks yield more challenges than the new building stocks do. The methods into circular buildings at large may not be sufficient nor suitable to convince any circular economy adoption in existing building sector^23–27^. Since there are many types of buildings and concepts for circular economy, our work consider all scales of CE implementation from materials viewpoint, to products and components, to existing building stocks, and to living built environments. The contribution of the study will enhance the policies and incentives to co-create and promote the implementation of circular economy for existing building stocks maximising sustainable impacts, while still minimising burdens on people and businesses.

## Review on current market barriers of building retrofit and renovation

Circular economy implementation actions in existing, aging built environments are mostly inspired by certain key outcomes including (i) minimised energy consumption (via deep renovation); (ii) prolonged service life of assets (through enhanced maintenance, reconstruction or retrofit); (iii) reduced wastes (by increasing recycling, reuse, repurpose, or rebrand); (iv) adaptation to climate change and external uncertainties (via retrofit or reconditioning); and (v) enriched structural condition and/or architectural aesthetics (through redevelopment or refurbishment). In fact, most actions through retrofit and renovation are incentivized to minimise energy usage (e.g. the annual deep energy renovation rate is 0.2% on average in the EU^20^), enabling either energy independence, or near zero energy buildings (NZEB), or zero energy buildings (ZEB), and even or energy positive buildings (EPB). The market for NZEB, ZEB and EPB is still emerging (especially for existing building segment) and therefore the existing collaboration structures are not yet able to demonstrate “successful” long-term collaboration apart from their contribution to exemplary NZEB new buildings and some extent of renovation projects. IEA EBC Annex 56 identified the current barriers in the renovation process (shining examples brochure available in^28^). Over the past decades, there have been many researchers across EU working on a number of projects related to building retrofit and renovation. In particular for existing building stocks, there are some key projects that could be instrumental to our study to define barriers to overcome the circular economy implementation to existing building markets. These projects include COHERENO, REFURB, RENERGY, and LOCARBO, which are closely related to the scope of this study.

In Europe, the COHERENO project (2013–2016) aimed to better understand the emergence of collaboration structures for NZEB renovation of owner-occupied single-family housing (SFH). IEE projects AIDA and PassREg both found organised study tours of best practice NZEBs to be an effective method of convincing municipal employees and decision makers the importance of including NZEB performance in design and tendering criteria for their building projects. AIDA project evaluated the major reasons for the municipalities against collaboration. The evaluation showed that two main reasons that hindered the municipalities to cooperate are: the tensed financial situation, where available budget is often needed for other investments and no budget seems to be left for investments in energy efficient buildings, and; the unwillingness of the municipalities to take action towards NZEB and RES. The experience has shown that energy efficient buildings have been considered as low or no importance issues for the communities. A further point, which was more often mentioned, was the circumstance that the municipalities did not have building projects, which were in line with the AIDA timeframe. Even when collaboration with a municipality was accomplished, many obstacles had to be overcome. Missing funds and unresolved financial questions represented the main obstacles to a successful collaboration. But also the missing personal awareness of the Mayor, insufficient/inadequate public policy instruments and other high-level officials as well as not established NZEB standards in the municipalities were bigger barriers. Very important for a successful collaboration was the on-going communication and active interaction, as well as the motivation of the municipalities, a flexible Integrated Energy Design (IED) work plan and an existing contact person at the right technical level in the municipality.

An important barrier that requires further work is the fact that the renovation market is principally supply driven rather than demand (consumer) driven^36–38^. In this sequence, the European’s REFURB project aimed to bridge the gap between supply and demand side. One outcome of this project is that despite the many experimental programmes and the informed work of certain design teams over the past decades, efficient buildings are still relatively rare and suffer from a lack of popularity, often because of a lack of publicity. Another outcome is a tool to tackle the complex interplay of these barriers through coordinated process organisation, innovation and optimization. European’s RENERGY project aim was to improve, by means of interregional cooperation, the effectiveness of regional development policies in the area of energy as well as to contribute to economic modernisation and increased competitiveness of Europe by turning urban spaces from energy consumers into energy producers. This project brings therefore to the community added value by developing a new and innovative approach and solutions in the field of energy at local and regional levels. The project faced the following issues: high up-front run capital costs of sustainable energy investments for a given amount of capacity. Policies that reduce these costs, such as loans, rebates, grants and tax incentives, could remove this significant barrier but are often lacking at the local level; lack of access to credit or funding for local governments, small and medium-sized enterprises (SMEs) and citizens to purchase or invest in renewable energy; lack of skills and experience and information at local governments with RES and decentralized energy system planning. This can lead to resistance towards positive change. Access to information, training, and exchanges can be highly beneficial in tackling this challenge, leading to a skilled workforce that can plan and implement policies, install and operate and maintain these energy systems; misperceptions on technology performance: Proven, cost-effective technologies may be wrongly perceived as risky by local government decision makers as well as by the public if there is little experience with them; numerous legal obstacles: Outdated laws (e.g. building codes, zoning laws, standards, permitting processes) can prevent, discourage or add expense to a RES project. Luckily, many municipalities have it in their power to remove such barriers and; need to transition to a smart energy system: Grid forecasting, smart grid features, and energy storage are not yet widespread, discouraging local governments fromaiming for higher RES targets and ambitious policies while they wait for the grid to be ready.

On the other hand, European’s LOCARBO project sought a change in improved implementation of regional development policies that incorporate actions to increase levels of energy efficiency including public buildings and housing sector. This is to be achieved by finding innovative ways for regional/local authorities to support energy consumers’ behaviour change. LOCARBO is unique by focusing its activities on bottom-up initiatives and mainly because of the approach to handle 3 thematic pillars (services, organizational structures and technological solutions) in a fully integrated way. The issues already addressed in the project are: many of Europe’s local/regional actor struggle with developing targeted, implementation oriented- policies addressing low carbon challenges. This holds particularly for energy wasting buildings irrespective of their ownership or their use. The implementation of these solutions is not targeted enough, related measures are often incidental and fragmented thus results lag behind expectations. Innovative stakeholder involvement measures are emerging, but they are not yet taken up at a significant scale. Specifically, local and regional authorities embark to find their role in these processes as coordination, planning, service provision, monitoring and feedback to policy making. Furthermore, there are various indications raising concerns regarding the reliability of Energy Performance Certificate (EPC) declarations and the quality of the works. The European’s REQUEST IEE Project focused on: identifying issues in respect to existing procedures; highlighting best practices for easy access to reliable EPC input data, delivery of improved quality of the works, as well as more effective compliance frameworks and raising awareness and engaging relevant stakeholders. This project pointed out the following outcomes: need for more support given to homeowners to help them move to the next stage in the customer journey after they have received an EPC outlining the recommendations and the partnerships between the supply and demand sides should be encouraged. The partnerships proved very effective in the pilots for improving communication and building trust at all levels—from project specific partnerships through to partnerships at the local, regional and national levels. Creating a partnership structure brings together a wide range of expertise, strengthening the multi-disciplinary insight from all parties and improving the ability for problem solving.

**Figure 2:**
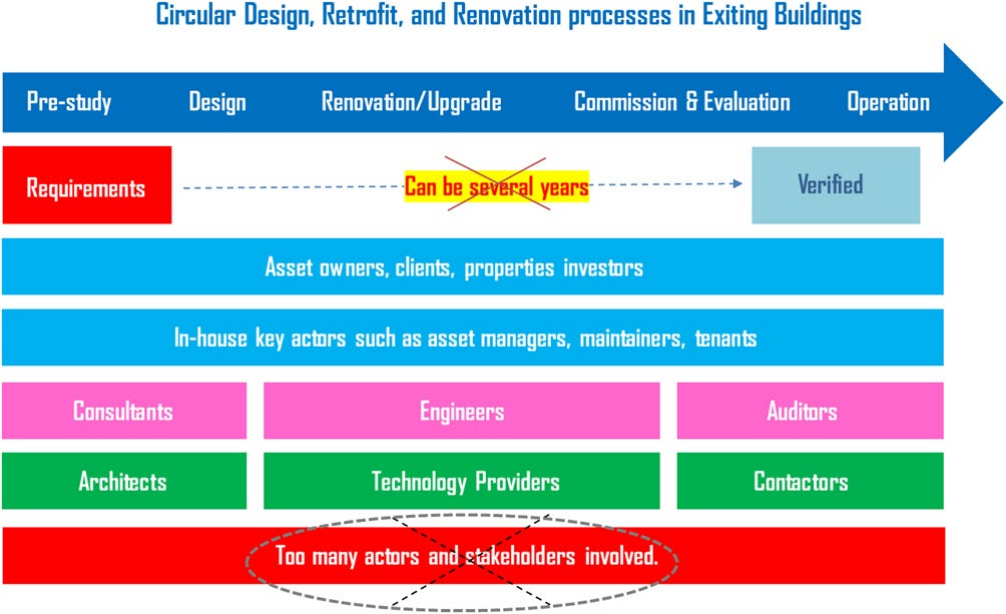
Typical retrofit and renovation process diagram.

In order to convince more building owners or influencers to go ahead with circular and energy-independent re-design, retrofit and renovation projects, it is very crucial that the forerunners are able to demonstrate that their projects actually succeeded in achieving the necessary quality for circularity and high energy performance. However, the building process usually starts with an initial concept followed by a number of different steps before finally reaching the operation phase. The time from concept to building site is usually long, perhaps several years. This extended design period involves a large number of different actors with varying levels of influence on the final energy performance of the building. This can make it difficult to actually achieve the energy performance that was set at the beginning of the process. In renovation projects, it can be difficult to compare energy performance and rate of circularity before renovation with the final built result, since the renovation in itself often alters the building in a variety of other ways, for example by improving the indoor environment and offering better possibilities to use the facilities. Levels of activity and numbers of users may be different: the building’s users may not be the same, and may not display the same energy behaviour before and after renovation. Particular consideration and guidance is therefore needed to ensure that the quality of the built result matches the ambition and actually achieves the desired energy savings. There is a challenge to eliminate the long period of time between pre-study and the operation stage (see Fig. 2) as well as the overlapping of actors and their ineffective collaboration.

During the last two decades, scientific and technological innovation has focused on the preparation of assessment systems and indicators to promote a basic level of energy efficiency. Objections can be raised concerning different interpretations of energy performance and systems boundary condition as it is proposed by EU norms and today’s evaluation methods. The current assessment has influenced design choices by awarding high or low energy certificate scores to only new buildings, of which their energy savings do not make a significant contribution to overall energy sustainability. It is evident that current approach has serious limitation in practice. One of the main objectives of this proposal is to focus on the new knowledge (theoretical and applied), the skills, the novel technologies and the competence that can support new working models that look beyond the scope of NZEB for new buildings, and that could lead to circular economy practices and adaptive and affordable energy-independent, energy-resilient solutions characterized by a wider range of integrated qualitative and quantitative retrofit and renovation innovations for the emerging market of existing buildings. The aim of this study is therefore to stimulate the development and integration of new knowledge, skills and competence at research and practical levels on the basis of multidisciplinary collaborations not commonly possible in a single standards research project. This study will bring together European sustainability researchers, energy harvesting experts, CAD/CAM professionals, building stakeholders and practitioners across a wide spectrum of disciplines to create unique and collaborative knowledge-based policies^29^. Through its participants and interviews, this study will seek to remove barriers and overcome scientific breakthroughs that exist in preventing progress for implementing circular economy practices towards regenerative growth.

**Figure 3:**
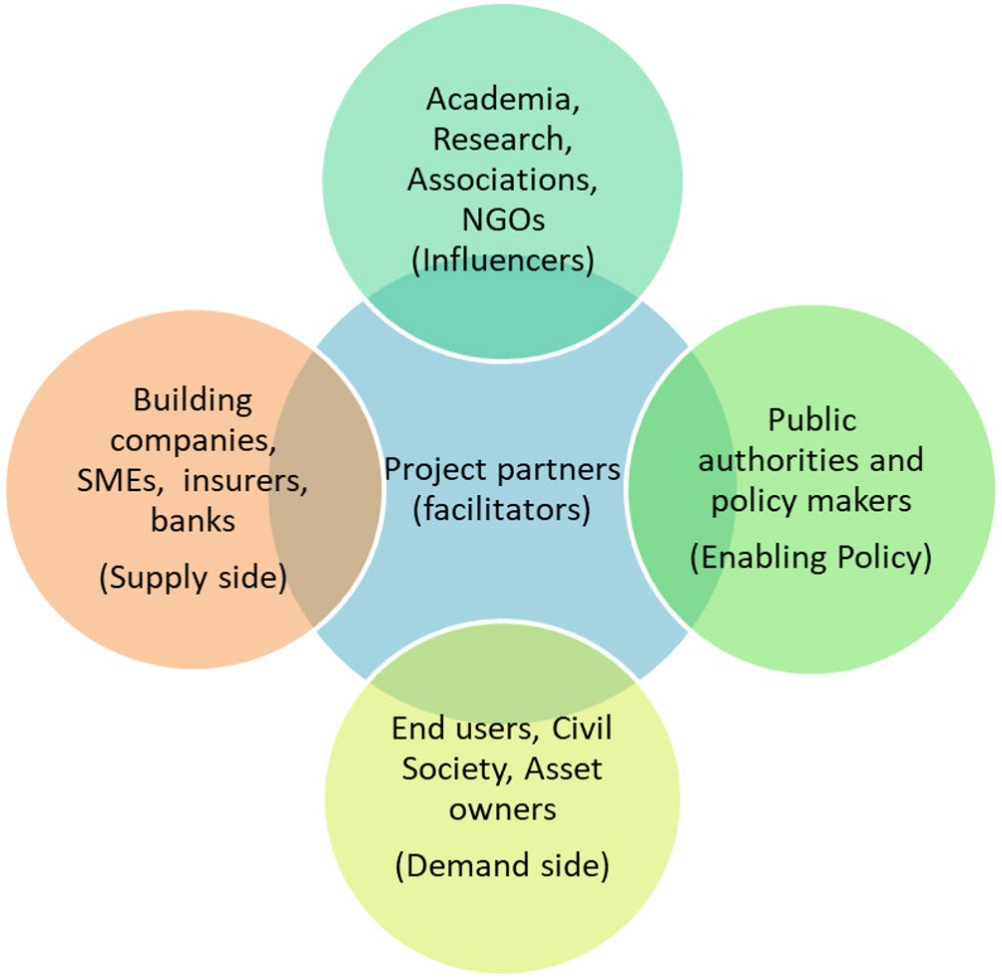
Circular economy target groups and interconnections.

This study embarks by engaging experts in CircularB project to preliminarily determine relevant stakeholders (see Fig. 3):

- Building end-users: building users, owners, and project developers aiming to construct, retrofit, and renovate either new or existing buildings (access to a ready to go platform with complete info for circular economy and renovation;
- Public authorities: government, municipalities (“official” platform to support circular re-design and renovation beyond NZEB, case study building, guideline for improved Energy Performance Certificate for ‘beyond NZEB’ building);
- Policymakers (guidelines for effective circular re-design, retrofit and renovation; and comprehensive EPC dedicated to both new and existing buildings over various stages of life cycle, guideline of best practices from case study buildings, publications);
- Building companies: construction companies and professionals such as engineers, architects, energy advisors, green building consultants (access to circular economy practice, re-design, retrofit and renovation approaches and strategies, training in ‘NZEB and beyond’, integrating the database in platform supported by municipalities, higher number of circualr re-design, retrofit and renovation works); product manufacturers (higher demand for insulation products, systems, ventilation systems and thermal energy storage units); energy suppliers (security of supply, lower energy network costs, reducing energy losses in the distribution networks, potential market for circularity);
- Banks and insurers (lower risks for the amortization of the credit and access to attractive split incentives).

From the above, it can be seen that the stakeholders can portray different levels of influences. The end users will mostly interact with facilitators (who are from both sides of supply and policy enabling). This implies that contemporary knowledge and understanding into stakeholders’ influences may be limited and there is a need to further enhance the insights into the stakeholders’ relationship and barriers at different stage of life cycles for existing building sector.

## State-of-the art concepts of circular economy practices for existing buildings

Achieving net-zero emissions will require a transformation of the global economy. It is important to note that energy-related emissions make up as much as 83% of CO_2_ emissions across land-use systems. Indeed, McKinsey^30^ stated that ‘*Effective decarbonization actions include shifting the energy mix away from fossil fuels and toward zero-emissions electricity and other low-emissions energy carriers such as hydrogen; adapting industrial and agricultural processes; increasing energy efficiency and managing demand for energy; utilizing the circular economy; consuming fewer emissions-intensive goods; deploying carbon capture, utilization, and storage (CCS) technology; and enhancing sinks of both long-lived and short-lived greenhouse gases*’^30^. The way for achieving a status beyond NZEBs of new buildings and of existing buildings is to promote the development, implementation, and automation of circular economy strategies by connecting market actors using Industry 4.0 Technologies via digital transformation and by carrying out a series of outreach activities. Innovative circular economy practices and alternative energy harvesting technologies can be included (such as the urban wind turbine successfully developed in WINNERCOST; solar energy developed by SME partners, etc.). Strategies to overcome market barriers to implement circular economy practices beyond NZEBs are through the simplification of the whole process of circular design, retrofit and renovation, to reduce the construction and commissioning stage and also by undertaking pre-simulation through a digital platform enabling the analysis of appropriate circularity strategies, energy performance, life cycle costing and attractive zero-emission/zero-pollution co-benefits.

**Figure 4:**
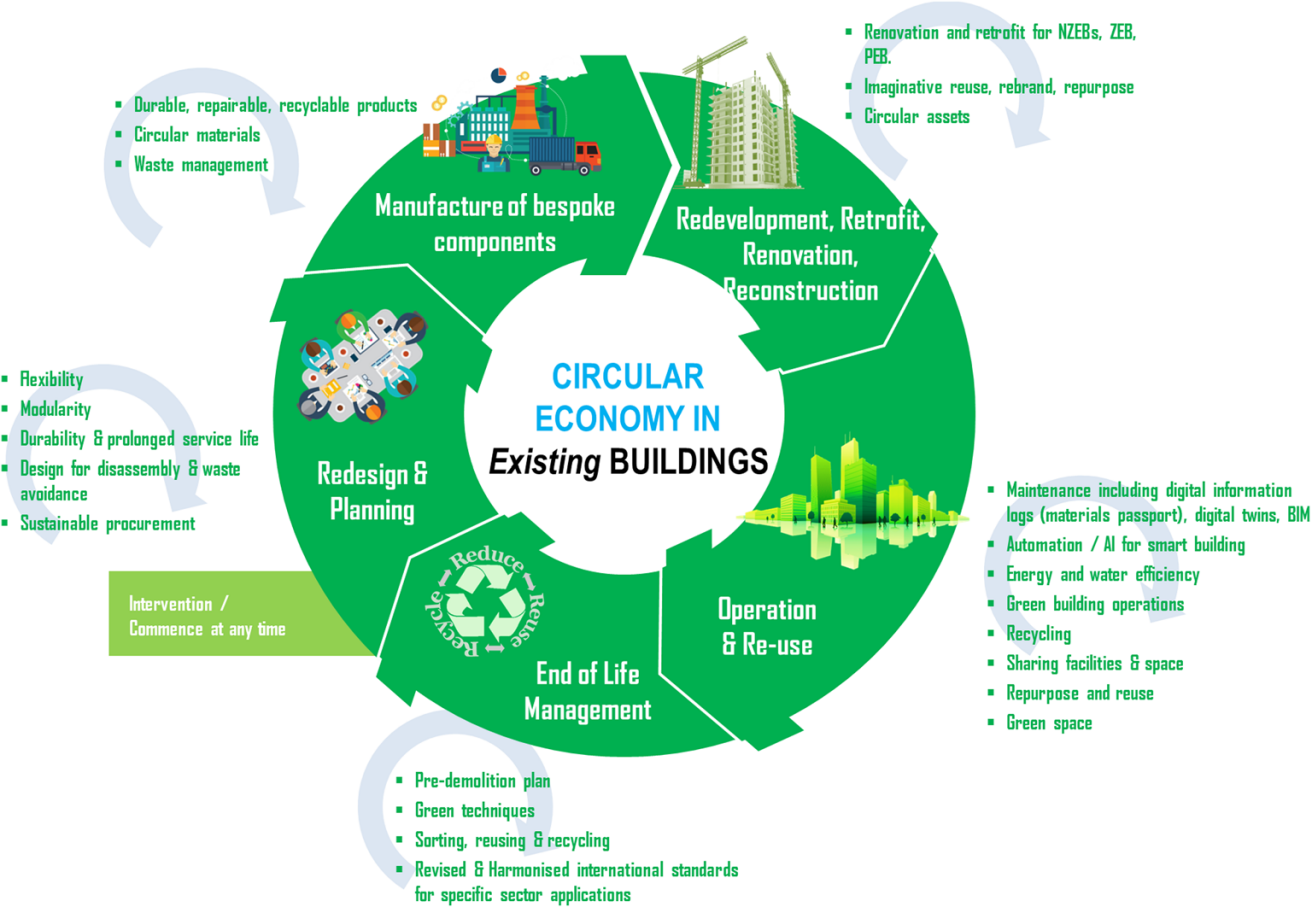
Circular economy perspectives and practices for the existing building stocks.

The circular economy generally implies an industrial economy that can be restorative by design; aims to rely on renewable energy; reduces, monitors, and eliminates the use of energy, water, carbon and toxic chemicals; and eradicates waste through careful design and planning^30,31^. As shown in Fig. 4, the circular economy perspectives for existing building stocks will consume much less resources and energy, while simultaneously being more carbon efficient and maximising waste reduction and management. From this, opportunities in zero energy and positive energy buildings can be identified and a flowchart with the stages for circular design, retrofit and renovation can be designed in order to reach a net zero. The traditional stages to implement circular economy concepts include:

**Table:** 

**Planning stage** Firstly, circular economy best practices, requirements and specifications for circular design, retrofit and renovation suitable to the buildings (i.e. residential or non-residential building) are identified. Available technologies, innovation and process for buildings energy are assessed. Market conditions are assessed, and required expertise for energy-independent, energy-resilient design, retrofit and renovation is identified, followed by the relevant technical stakeholders. Furthermore, other stakeholders that are often neglected in the retrofit and renovation process for existing buildings or only required for a specific stage, namely architect, insurers, energy and financial advisors, are to be considered^32,33^. These actors play a significant role in circular economy concepts. As known, even NZEB is still not widespread for existing building sector (which is the largest fraction of built environments), therefore the intervention of a facilitator specialized in energy and buildings can be key to the success of a project. The facilitator’s role is to help achieve the definition of objectives, integrating energy selection criteria into the specifications and to make sure that stakeholders and contractors are appropriately implementing the chosen approaches and technical solutions to achieve the desired targets. They can ensure that the original intent of the project is maintained despite any difficulties that may be encountered. A facilitator will also encourage and smooth exchanges between professionals and contractors. In parallel, attractive financial tool is substantial to encourage existing home owners and building managers to undertake retrofit and renovation towards net zero The decision making in circular building design, retrofit and renovation is also influenced by a number of non-technical stakeholders such as: public authorities (national, regional, municipal, local), in their capacity as building owners, as enablers/ facilitators, as policymakers, or as financers; building owners, either as landlords or as owner – occupiers; industry players (suppliers, contractors, energy service companies); professionals (architects, engineers, building managers, surveyors); insurance and financing entities (public or private); occupiers and end-users. The selection of the actors to integrate in energy-resilient building concept is accordingly to their expertise, responsibilities, proven experience and willingness of adopt new strategies in circular design, retrofit and renovation to integrate later on our trustable database and digital twins. The home owner/end user is to integrate the team during the whole process and to actively participate in the decision process. One of the biggest barriers in design, retrofit and renovation process is the collaboration between all actors and with the building users. In general, there is a lack of interaction between stakeholders (silos in authorities, dispersion of decision making) at all levels. Amongst owners and end users the main challenges are related to uncertainties and a lack of trust in technical and economic information related to the renovation schemes offered to them. Potential solution pointed out by EU Circularity Action Plan are new, inclusive and empowering collaboration models at all levels: overcoming silos in regulations and policymaking; “co-maker” schemes in industry (i.e. close partnerships between different manufacturers) that can secure cooperation beyond single projects; creating alliances at the district scale, giving each stakeholder a key role in a joint planned approach. In this alignment, a collaboration scheme between all parts is developed. This kind of shared holistic approach with clearly defined models of cooperation helps provide security of investment and ensures the “buy-in” of stakeholders^34,35^ In order to convince more building owners to go ahead with circular economy concepts, energy-resilient design and renovation projects towards net zero, it is crucial that the forerunners are able to demonstrate that their projects actually succeeded in achieving the necessary quality towards net zero (e.g. for high energy performance beyond NZEB, ZEB and/or EPB). Various forerunners partners are therefore to be actively involved in the planning step and to guide in all stages. Also, involving the local municipalities will overcome this barrier, namely by creating an “official” platform using living labs (based on demonstration buildings) with trustable databases as well as regular workshops and conferences to promote discussion and share experiences from owners and stakeholders’ perspective. In terms of technical solutions, different design, retrofit and renovation measures based on circular economy concepts are investigated according to the type of buildings, state of the building, location, etc Cost optimality analysis is performed to select the best scenarios against meta-operational conditions throughout the life cycle. The methodology used in the calculation follows the EPBD, ISO 14040 and ISO 52016^36^. It builds further on methodological work carried out in the IEA EBC Annex 56 project on Cost-Effective Energy & CO_2_ Emissions Optimization in Building Design, Retrofit and Renovation, in particular with respect to the framework for comparing measures improving the circular and energy performance with reference measures, which would be necessary just to restore functionality of the building elements, and for taking into account lessons learnt related to the balance point between renewable energy measures and energy efficiency measures. Additional foci can be given to the following solutions: super insulating materials (SIM) for specific renovation details, nature-based solutions (such as planted roofs and elements of the built environment), high performance windows and glazing systems, ventilation systems and products especially for renovation compact and affordable storage systems (thermal energy storage systems), thermal and photovoltaic panels, aluminium and PVC windows an d control technologies, urban wind energy harvesting technologies, and advanced sensing and automation to enhance net zero concept^36^ The advanced sensing for energy performance in built environments and the potential of sharing renewable energy systems (RES) between buildings can also be considered in the planning stage. RES installations often come across problems with ‘cross-ownership’, or the superposition of rights on land and infrastructure. Not all decisions and rights are handled at the same political level (local, regional, national, EU) which can hamper integration and implementation of cross-sectorial solutions. This will be fully considered in the analysis of integrating RES. For the suitable measures, life cycle costing (LCC) and carbon footprint is undertaken. Building and home owners frequently do not have a structured way to obtain all the necessary information namely a clear financial and benefits plan, hence attractive incentives can be also developed and integrated within the digital technology platforms such as BIM, digital twins, sensor placements, data science, and artificial intelligence The interconnected suite among energy-independent methods and circular economy strategies, cost optimality, co-benefits, LCC and incentive model can be developed to give home owners and building professionals a way to easily assess and compare different design, retrofit and renovation strategies for a given building, combining energy calculations and cost calculations with a flexibility and a depth which makes it unique and vividly visual. This will allow a dynamic interface, which allows making a first assessment with a minimum amount of input parameters, while providing the option to adapt calculation parameters up to a high level of detail. The integrated platform will allow calculating energy performance, greenhouse gas emissions and an economic ex-ante evaluation over the entire life-span of design, retrofit and renovation measures. The suite is to comprise a comprehensive database of empirical techno-economic characteristics of building design elements and renovation measures to provide meaningful results. The platform can show transparently costs, energy use and greenhouse gas emissions of different design and renovation packages. It will simplify the complex energy properties of a building to an extent that make economic and energy assessments accessible and understandable for building owners, while still having sufficient precision to provide meaningful results for other stakeholders. The platform can be customizable for different country contexts, in particular related to climate data, cost data, emission factors and primary energy factors of energy carriers, and other country-specific parameters. There are differences in national calculation procedures for determining circular materials, water consumption and energy needs in building and for assessing energy performance in building, despite the common norms and regulations. These differences can be taken into account in the calculation engine For each renovation measure, the user can differentiate what part of the costs is to be associated with the circular design and renovation, and what part of the costs is to be considered as costs not related to the circular economy concepts of the design and renovation measures, but rather to other reasons. For example, if a new window is installed, the user can give a weight to the co-benefit of noise-protection by estimating that about half of related costs are to be considered as not energy related. The costs, which are accepted for other reasons than improvement of energy performance, are deduced from the indicated costs of the measures. This way, a more appropriate assessment of energy related costs and impacts is made, and the users own valuation of co-benefits is taken into account. By providing such a way for building owners to take into account quantification of co-benefits, they are encouraged to consider as cost-effective measures also measures that without taking into account co-benefits are often not cost-effective as the installation of a new window
**Execution stage** Pre-simulations can be performed for the buildings using a digital platform. The best scenarios are to be identified and applied. The circular building data can be continuously collected to update the automation (e.g. materials, water, wastes, energy usage control in each part of the building). It is crucial to overcome current market barriers towards net zero, and therefore create awareness of the barriers in both sides. Collaboration needs to be established among academia, industry, municipalities and SMEs in order to implement circular economy concept successfully. The technical and quality assurances of the circular design, retrofit and renovation measures should be guaranteed and circular economy checklists should be prepared to assess this process^37^. All activities are crafted to well support the successful delivery of circular economy implementation
**Commission stage** It is important to ensure the monitoring platform for compliance purposes in order to validate the results obtained of the digital suite or circular economy goals, and hence there will be pressure for a correct assessment of the reliability of the results. A repetitive process should be performed in order to validate the outcomes of the circular economy concept application. The monitoring itself can be carried out through sensors, routine inspections, and smart meters to monitor different parameters affecting water and energy consumption, waste management, and/or internal comfort. These data can be collected to update the artificial intelligence model for smart building automation that will drive the race to net zero. Moreover, users experience is to be evaluated through interactive activities and surveys. The duration of the monitoring period is also an issue to consider among stakeholders. In case of discrepancies, a refinement procedure (i.e. enhanced maintenance, retrofit, redevelopment) should be performed and the circular economy framework for the building should be updated accordingly A focusing attention is needed more broadly on the multiple benefits of circular upgrade, retrofit and renovation. Disconnect from the “kWh” and “payback” rationale, towards values that are closer to consumers e.g. definition of home quality standards, addressing intellectual and emotional issues, health benefits. Recent reports by the International Energy Agency (IEA) and by the United Nations Environment Programme (UNEP DTU) provide hard evidence that energy renovation offers many benefits to existing buildings’ owners beyond cost savings^38^. Again, although monitoring is an important tool in evaluation of an energy renovation, it should not only measure energy performance (kWh/m^2^) but also: the indoor environmental quality (e.g. temperature, air quality, and visual comfort), airtightness, the rebound effect, the weather conditions, occupancy (internal gains, building use) and user experiences. This stage will also allow to identify and list the co-benefits that can result from successful circular economy implementation and to also be applied within the EPC. Also, it will allow accurate drafting a guideline for circular design upgrade, retrofit and renovation measures for existing building stocks and guide asset owners in the further interventions. Advertisement experts are to join the process from the beginning and develop clear and interactive campaigns to promote circular economy concepts. This intends to overcome communication and awareness-raising that are constant requirements at all levels

Although the circular economy concepts and applications to existing building stocks can be realized in practice, the adoption of those measures is unsatisfactory and may not help to achieve the net zero goal by 2050. This is because technological solutions alone cannot resolve the global climate challenge. There is a need to engage societal and indiviual wills to make it happen. As such, the inter-relationship among stakeholders, the influencer and strategies to overcome the barriers are critical to convince the existing building sector to implement circular economy concept towards and beyond net zero. The insights into the influence and inter-relationship among stakeholders of existing building stocks have not been thoroughly identified. Thus, this study will determine and compare the influence and inter-relationship among stakeholders, and will highlight the barriers to implementing circular economy concepts in existing building stocks across Europe.

## Methodology

**Figure 5:**
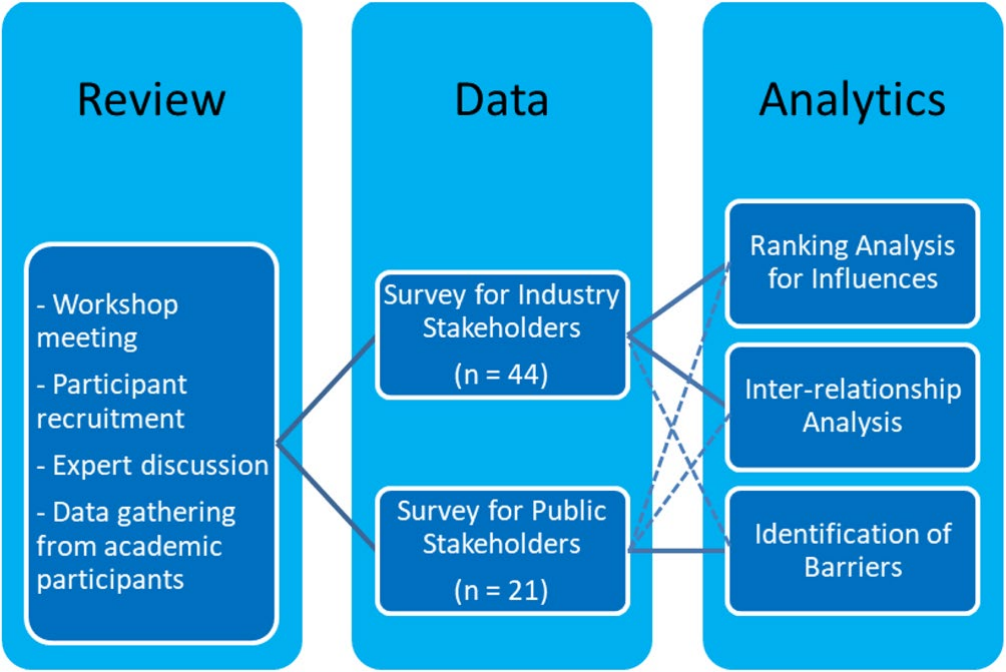
Research methodology.

The research adopted a qualitative and on-field approach via semi-structured interviews and dialogue with different stakeholders and operators of the existing building stocks’ value chain (see Fig. 5). The non-personal data was collected anonymously without withholding person information. All respondents had given consent for data collection. The data requested in this study was collected and processed by the researchers in accordance with the provisions of Regulation (EU) 2016/679 (the General Data Protection Regulation, GDPR) and all other applicable EU and UK privacy and data protection legislation. This study is GDPR compliant and has been approved the University of Birmingham’s IRB. The research tasks will determine current policies and practices of circular economy at the building level and associated initiatives, influence, inter-relationships, incentives and barriers. The investigation by semi-structured interviews were conducted across 5 different European countries including Finland (FI), Germany (DE), Latvia (LV), Türkiye (TR), and United Kingdom (UK). These counties constituted intensive research into circular economy in built environments and portrayed the diversity of context, enabling the authors to investigate a variety of dynamics and impacts in the geopolitical and practical fields.

The semi-structured interviews were preceded by a critical literature review and a desktop study. The review took into account the workshop meetings and discussions with 21 experts and researchers in CircularB project (WP2). These expert interviews have been considered to be substantial for qualitative research^39,40^. In general, specialised expert opinions tend to converge after 20 interviews^41,42^. In addition, the data from their previous research has been collected to understand the trend and current issues with respect to circular economy implementation in their countries. The in-depth analysis of the data can later point out essential questions for different stakeholders.

On the basis of the initial literature review and desktop study, the stakeholders have been grouped by commercial purpose into: industry stakeholders and non-industry stakeholders. Table 1 defines the detailed stakeholders of existing building sectors across Europe. Consequently, the survey through the stakeholder interviews has been conducted to further gain the crucial information, for later data analyses including (i) ranking for influence, (ii) inter-relationship correlation, and (iii) barrier identification.

**Table 1:** Stakeholders of existing building sectors classified by stage of life cycle derived from initial expert discussions and workshops with CircularB project members/participants across Europe. This has been correlated with Europe’s industry waste^43^.

Stage of life cycle	Who are stakeholders for residential and non-residential buildings?	What are contributors to CO_2_e?	What are emerging CE strategies/tools/mechanism in your country?	What are CE implementation barriers and challenges?
Planning and Design	Owners/Investors Financial institution Local councils/urban planners Architects/Engineers	Energy	Deep renovation Urban wind Photovoltaic technology (PV) Solar thermal energy LCA/Digital twins/BIM Design for reuse/repurpose	Living cost Financial burden No incentives to improve No governmental directives Risk-averse attitude
Construction /Retrofit /Renewal /Refurbishment /Renovation	Construction companies Manufacturers Engineers Experts/researchers	Materials (1. Concrete; 2. Steel; 3. Plastics) Machineries Water Waste Energy	Material circularity (e.g. material passport/BIM) Component circularity (e.g. digital twins) Renewable energy grid Waste reduction (e.g. BREAM)	Limited options of technologies No incentives No legislation/standards/specification
Operation/Use	Asset owners Residences (dwellers) Maintainers Experts/researchers	Water Waste Energy	Resource Efficiency Energy efficiency Waste management	Limited methods for service life assessment Human behaviours No incentives
End of life	Asset owners Demolition companies Waste managers Experts/researchers	Waste (building materials, electrical appliances; furniture) Energy	Material circularity Net zero target Material recycling	Toxicity Uncertainties Limited recycling technologies Limited Standards and specifications
Intervention Phase (dealing with residues)	Exporter of wastes Environmentalists	Residuals	Energy recovery	Landfill Toxicity

In order to extract different scales and dimensions related to the influences, inter-relationships and barriers of the circular economy implementation in the existing building sectors (e.g. deep renovation, retrofit, energy harvesting, energy independence, etc.), open-ended questions have been established and divided into 3 main themes:

- influence among stakeholders: this aspect is critical to understand decision making processes, soft and hard power, obligations and incentives that could promote circular economy concepts. This can represent a buttom-up trigger or appeal for competitions and attraction towards circular practices;
- inter-relationships among stakeholders: the insights will help to determine the value chain of circular supply chain network, which identifies the dynamics of who, what, when, how and why for any decision towards circular practices to be made.
- challenges and obstacles to implement circular economy concepts to existing building stocks: the insignts into these facets are the key enabler for accelerating the adoption of circular practices. There are a number of non-flexible or outdated regulations, risk-averse standardization and specifications, incomplete tools and technologies, and inadequate financial support mechanisms; all of these could discourage the implementation of circular economy concepts.

For each theme, some questions have been defined as shown in Table 2. The questions are designed to reflect the role and responsibility of associated stakeholders. The questions can introduce a dialogue with each participating interviewee from each European country of focus. Each interview or meeting has been conducted with one stakeholder at a time, in person or via Zoom/Team call, and lasted a minimum of 0.5 h to a maximum of 1.5 h. Semi-structured interviews have been conducted with different stakeholders of the existing building value chain from five countries. The interviews consist of open-ended questions related to the themes. The sequence of questions and the style of conversation can change with the responders, to create a safe and confidential environment of knowledge exchange and to capture the viewpoints raised by the interviewees.

**Table 2:** Overview of the questions used in the interview.

Theme	Stakeholders	Example of Key Questions
Influence among stakeholders	Owners/Investors/Residences Financial institution Local councils/urban planners/Decision Makers/Environmentalists Architects/Engineers/Designers Constructors/Manufacturers/Maintainers/Demolition	For existing building stocks, who make the decision on CE implementation? What is your role in CE practices? Who or which stakeholders provide the incentive for CE practices? Who or which stakeholders influence you to consider CE? Can you rank the top 5 of the most influencers on CE implementation?
Inter-relationships among stakeholders	Owners/Investors/Residences Financial institution Local councils/urban planners/Decision Makers/Environmentalists Architects/Engineers/Designers Constructors/Manufacturers/Maintainers/Demolition	Who can influence you on the CE practices? Whom can you influence on the CE practices? What is the CE value chain around you? How do you build relationships among your value chain to implement CE practices? Can you tell me about a time when you developed and maintained a relationship with a business partner and explain how you did it?
Challenges and obstacles to implement circular economy concepts to existing building stocks	Owners/Investors/Residences Financial institution Local councils/urban planners/Decision Makers/Environmentalists Architects/Engineers/Designers Constructors/Manufacturers/Maintainers/Demolition	Which current policies promote CE in your country? What are obstacles and challenges to CE implementation? Are there any incentives for CE implementation (i.e. reuse, repurpose, deep renovation, retrofit, energy harvesting, energy indepence)? Can you help list and rank Top 5 challenges and obstacles to enable CE practices?

The responders are chosen through a sample of respondents selected by the authors and based on their knowledge and expertise of topic under investigation. Through snowball sampling method, the aim of selections is to identify the best representative stakeholders of public and private sectors currently involved and interested in circular economy. The responders selected have already relevant experiences for example, ‘Smart Campus’ in the UK. On this ground, the snowball sampling is suitable for this study. In fact, the random sampling of respondents is not suitable for this study since the knowledge of argument is not diffuse across all stakeholders. The randomization could actually cause bias and may fail to highlight virtous paths and critical burdens to be overcome.

In this study, totally 65 stakeholders (FI: 6; DE: 9; LV: 6; TR: 35; UK: 9) were available for an interview (nearly all stakeholders were contacted and interviewed either in person or virtually, or approximately 95% success rate). The number of stakeholders is enough to involve all key players across six countries. Anonymity has always been ensured to all respondents to create safe, fair and inclusive environment. Each stakeholder received an interview guide and one of the authors would conduct all the interviews.

### Cross-comparison analysis

**Figure 6:**
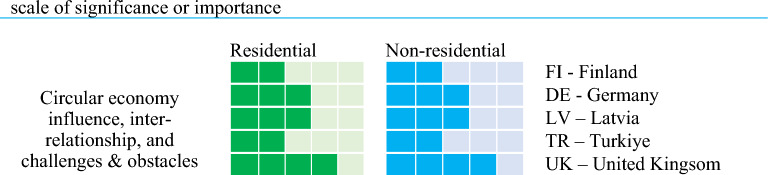
Example of qualitative methodology for cross comparison analysis.

To analyse the data collected from the interviews, a cross comparison analysis has been investigated. Cross country comparisons make it possible to obtain insight across multi-national scopes and scales. To conduct the cross comparison analysis, the interview data has been rearranged to form a matri of influence vs stakeholder inter-relationship. The analysis of matrix will help assess the policy impacts and incentives for circular economy practices. The application level is represented by the number of boxes ticked as shown in the cross comparison diagrams (see Fig. 6): 4–5 boxes indicate the highest/maximum level of application or influence; 2–3 boxes represent a medium level; and one box reveals the lowest or minimal level of application or influence; and no box ticked indicates ‘not yet applied’.

## Results and discussion

### Influence among stakeholders

**Figure 7:**
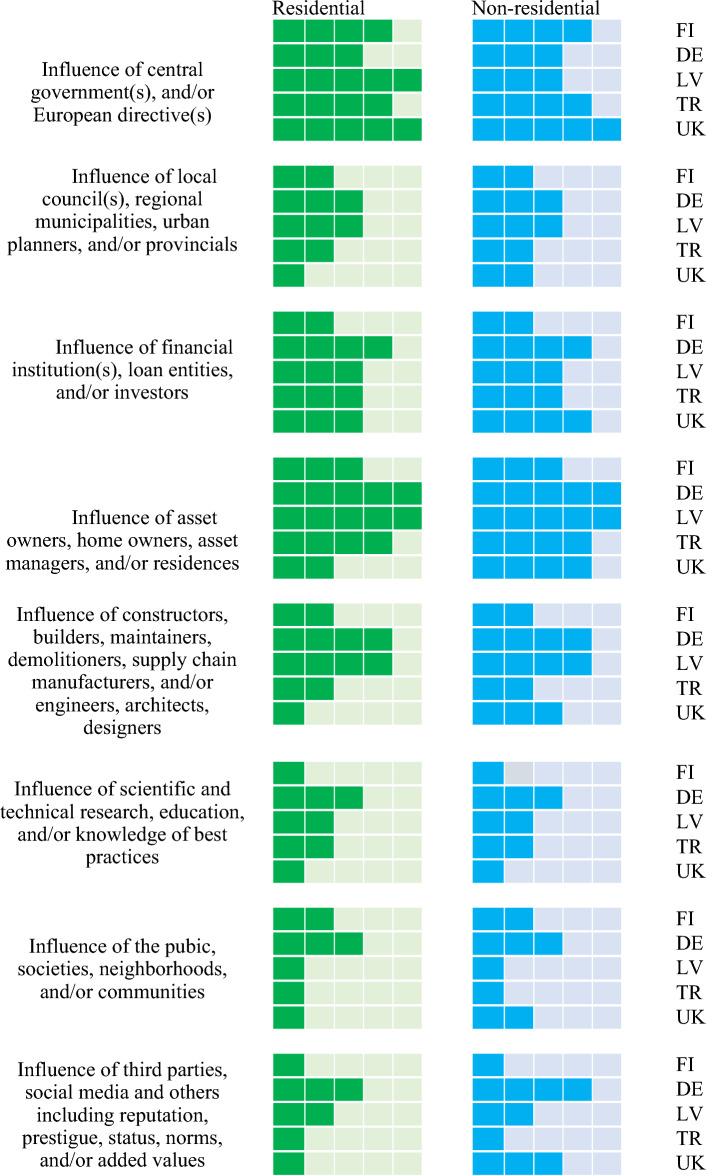
Cross comparison of circular economy stakeholders’s influences.

A primary topic related to circular stakeholder engagement is the influence among stakeholders involved in circular practices for existing building stocks. Derived from the extensive expert and stakeholder interviews, Fig. 7 illustrates the influences among stakeholders perceived by circular economy practitioners. Based on Fig. 7, the influences among stakeholders can vary from country to another. Based on over 60% of significance scale, the top three most influencers are (i) central government and European union, (ii) asset owners and managers; and (iii) financial institutions and investors. Most experts and stakeholders have pointed out that these influencers play a key role in circular economy implementation, which were fully agreed during the interviews. It is noted that the central government and European authority have a role to play to influence the public on this aspect. They can devise inventives, legislations, enforcements, and penalties that strongly guide the decisions of other key actors or agents (e.g. asset owners, investors, business) to implement circular practices.

It is also interesting to observe that the influences from social medias, third parties, neighbourhoods, local communities, scientific research and educational institutions are not very pronounced. Despite the strong presence of climate emergency on social media, televised broadcasting, documentaries and online plaforms, the actions to reduce global warming to meet Paris Agreement are unsuccessful. This is evident by science-based targets where merely 20% of global businesses support the action towards the reduction of greenhouse gas emission. In addition, some experts and key stakeholders clearly pointed out during the interviews that the access to relevant research and education is relatively poor in many counties. Research outcomes, convinceable insights and key outputs cannot be accessed by the decision makers such as asset owners or asset managers. Such the lacking was stressed by home owners and various actors though circular economy supply chains. At the same time, there has not been any peer pressure nor peer aspiration within or by local communities and neighborhoods to implement circular economy concepts to existing buildings. In addition, they would need to carry out due diligence prior to consider random social media posts/blogs. These have been some of the concerns resulting in the weak influences from these actors.

### Inter-relationship among stakeholders

**Figure 8:**
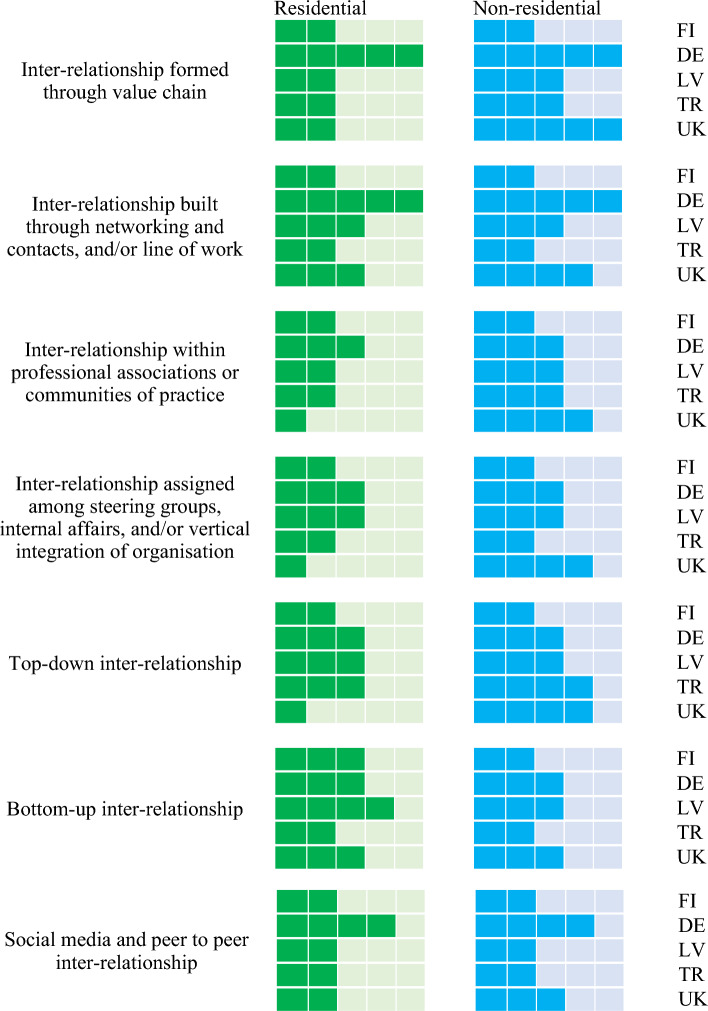
Cross comparison of circular economy stakeholders’ inter-relationships.

Figure 8 demonstrates the inter-relationships among stakeholders synthetized from the expert and stakeholder interviews. This aspect is relatively complex since stakeholders are interconnected and influenced. Experts’ opinions are analysed and reaffirmed to identify the connections between stakeholders involved in their practices. Based on Fig. 8, the inter-relationships among stakeholders are pronourced differently from a country to another. It is clear that the high degree of inter-relationships (above 50% of significance scale) are formed through value chain and industry network in the non-residential building sector. This is because each stakeholder does not often connect with the other stakeholders outside their own supply chain network due to their time contraint. The implementation of circular economy practices tend to be a top-down inter-relationship rather than bottom-up approach. This is because the key influence is often by the governmental policies and legislation. The communications tend to be direct down from the top layer of value chain network. A few home owners highlighted the key insights that there is a lack of financial support and inter-relationship across the value chain, making it difficult to pursure the renovation or to retrofit their aging homes. Based on the stakeholder interviews, most home owners often focus on the upgrade or renovation of their aging homes to improve energy efficiency and reduce energy costs. They often discussed with local contractors and after a few round of discussions about circular economy techniques or methods (e.g. solar panels, thermal insulation components, waste management, wall or roof retrofit, façade renovation, etc.) and associated costs, they often discontinued the plan to upgrade their homes. This was mainingly due to the financial constraints and the perceived low value for money.

It is also important to point out that various asset owners and investors still have some concerns about the quality of parts, products, and components made from either wastes or recycled materials. Such the concerns have not been well addressed by the inter-relatonships among value-chain stakeholders and industry or professional networks. The experts pointed out that there were not many customers who wish to use second-handed or reclaimed components or products, which might increase depreciation and potentially reduce the life span & future value of their assets. In addition, the community of practices, professional associations and social medias do not gain sufficient ‘market pull’ that can build inter-relationships and sufficiently convince asset owners and key decision makers to adopt CE strategies and tools such as lifecycle perspectives, recycled products, green quality certification, and so on. Based on the expert and stakeholder interviews, there is infufficient market place or exchange platform for recycled or reclaimed materials, components, and/or products. This was due to the lack of standardization for certification, incentives, and accounting tools to record and report the circular practices and their costs/benefits.

### Challenges and obstacles to implement circular economy concepts to existing building stocks

**Figure 9:**
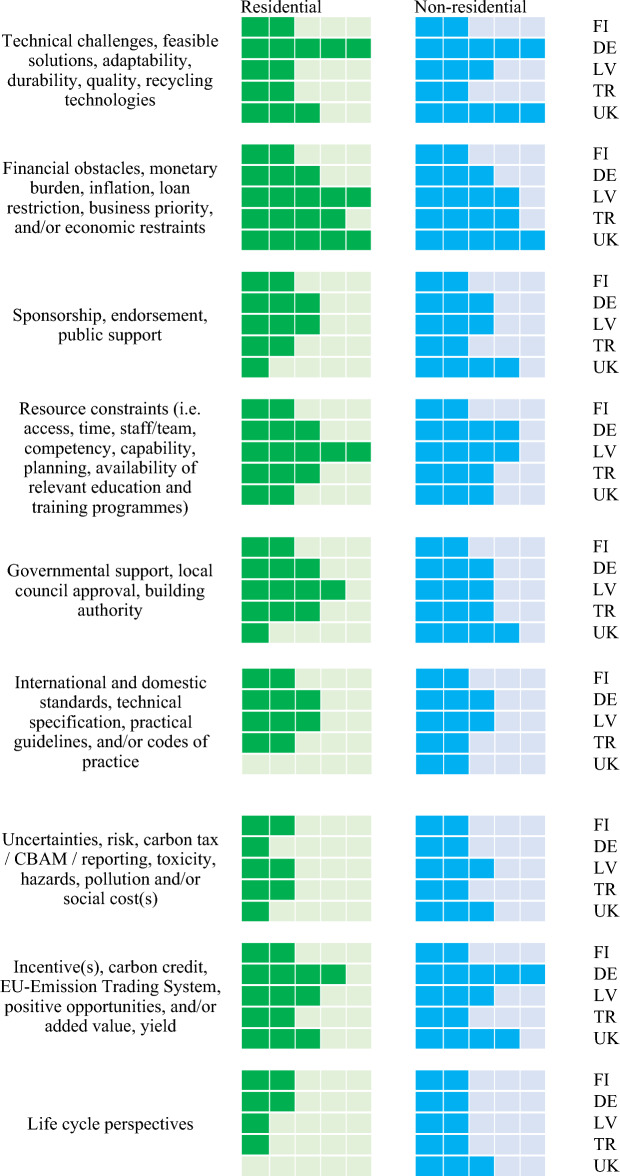
Cross comparison of challenges and obstacles to implement circular economy practices faced by stakeholders (con’t.).

Figure 9 provides the significance of challenges and obstacles to implement circular practices to existing building stocks faced by industry practitioners. By delving into the expert and stakeholder interviews, the most critical challenges and obstacles faced by asset owners, decision makers, and other key stakeholders include: (i) financial burden; (ii) resource constraints; (iii) governmental support and enforcement; and (iv) technical challenges. Various experts and stakeholders have stressed the importance of knowledge sharing and educational programs with respect to circular economy and sustainability, which can alleviate the challenges and eliminate some concerns that decelerate the adoption of CE practices. They addressed further that new economic activities (e.g. second-handed material exchange platform) could be incubated from further circularity awareness and knowledge dissemination. In addition, asset owners and investors suggested that financial incentives together with clear and committed governmental policies and enforcement be enacted to mitigate inherit barriers to implement circular economy practices. Certain valorisation value and virtuous circle systems such as carbon credit, carbon tax, auditing system, digitisaiton, BIM, etc. will be highly instrumental to the successful pathway to transition to net zero.

## Conclusions

Buildings account for around 39% of global carbon dioxide emissions. In addition, buildings consume around 40% of all the energy produced, cause over 35% of the EU's total waste generation; and account for about 50% of all extracted (fossil) materials. Based on this statistics that the building industry is a key sector required to be revolutionized to meet national and international net zero targets. Recently, CircularB Project funded by European Cooperation for Science and Technology (COST) has been devoted to co-create synergies of digital transformation solutions, data-driven & complexity science and technical capabilities towards achievable, affordable and marketable circular buildings and potentially enabling further circular economy solutions for both new and existing buildings. However, it is clear that technical solutions alone cannot resolve the global issues. This paper delves into socio-technical investigations to derive new insights into the stakeholders’ influences, inter-relationships, and obstacles in the implementation of circular economy concepts on existing building stocks. It is important to note that, based on EU database, existing building stocks represent over 90% of whole building assets. This outcome of this study will therefore help put the circular economy agenda on the fast track to net zero transition. A robust critical literature review of key documentations such as articles, standards, policy reports, strategic roadmaps, and white papers has been conducted. The key stakeholders of existing building stocks at different stages of lifecycle can then be identified. It is also clear that there has not been any research devoted to this particular aspect in depth. Our extensive expert interviews with relevant stakeholders and decision makers draw new insights across all scales of circular economy implementation, including:

- With over 60% of significance, the top three most influencers include (i) central government and European union, (ii) asset owners and managers; and (iii) financial institutions and investors.
- The top influencers can significantly guide the decision towards the circular economy implementation.
- The high degree of inter-relationships among stakeholders are built through value chain and industry network.
- The top-down inter-relationship plays a key role in the implementation of circular economy practices.
- The most critical challenges and obstacles in the implementation of circular economy include: (i) financial burden; (ii) resource constraints; (iii) governmental support and enforcement; and (iv) technical challenges.

Our new research findings demonstrate the essential need for harmonized actions and tactical/pragmatic policies promoted and regulated by the European Commission, national and local governments who can dominate the influence, promote inter-relationship, and overcome the barriers towards circular economy much more effectively. The financial incentives and governmental enforcement (via policy, legislation, or directive) are reported to be the most critical obstacles that required an urgent need for consideration. Open-access knowledge sharing and educational programs will be instrumental to accelerate the adoption of circular economy practices in the existing building sector.

## Data Availability

Informed consent was obtained from all respondents. There is no experiment involving human participants. Data available on request to the corresponding author.
